# Therapeutic Potential of Hyporesponsive CD4^+^ T Cells in Autoimmunity

**DOI:** 10.3389/fimmu.2015.00488

**Published:** 2015-09-22

**Authors:** Jaxaira Maggi, Carolina Schafer, Gabriela Ubilla-Olguín, Diego Catalán, Katina Schinnerling, Juan C. Aguillón

**Affiliations:** ^1^Programa Disciplinario de Inmunología, Immune Regulation and Tolerance Research Group, Facultad de Medicina, Instituto de Ciencias Biomédicas, Universidad de Chile, Santiago, Chile; ^2^Millennium Institute on Immunology and Immunotherapy, Santiago, Chile

**Keywords:** tolerogenic dendritic cells, T-cell anergy, regulatory T cells, hyporesponsiveness, immunotherapy, autoimmune diseases

## Abstract

The interaction between dendritic cells (DCs) and T cells is crucial on immunity or tolerance induction. In an immature or semi-mature state, DCs induce tolerance through T-cell deletion, generation of regulatory T cells, and/or induction of T-cell anergy. Anergy is defined as an unresponsive state that retains T cells in an “off” mode under conditions in which immune activation is undesirable. This mechanism is crucial for the control of T-cell responses against self-antigens, thereby preventing autoimmunity. Tolerogenic DCs (tDCs), generated *in vitro* from peripheral blood monocytes of healthy donors or patients with autoimmune pathologies, were shown to modulate immune responses by inducing T-cell hyporesponsiveness. Animal models of autoimmune diseases confirmed the impact of T-cell anergy on disease development and progression *in vivo*. Thus, the induction of T-cell hyporesponsiveness by tDCs has become a promising immunotherapeutic strategy for the treatment of T-cell-mediated autoimmune disorders. Here, we review recent findings in the area and discuss the potential of anergy induction for clinical purposes.

## Introduction

Effective peripheral tolerance mechanisms are required to eliminate circulating autoreactive T cells and thereby prevent undesired immune responses against self-antigens. The key players in this process are dendritic cells (DCs) that induce tolerance by different control mechanisms such as T-cell deletion, generation of regulatory T cells (Tregs), and/or induction of anergy (1–3). This tolerogenic role of DCs has aroused the interest for their *ex vivo* generation and their application as therapeutic tool to restore tolerance in autoimmune conditions or allergy.

Interaction between DCs and T cells occurs through three independent signals: (i) recognition of peptide-MHC complexes presented on DCs via specific TCR on T lymphocytes, (ii) binding of costimulatory molecules expressed on DCs to their respective receptors on T cells, and (iii) polarizing cytokines secreted by DCs (4). When presentation of antigen peptides by DCs occurs in the absence of costimulation, T cells become anergic (5). Anergy is a hyporesponsive state that retains T cells in an “off” mode under conditions in which immune activation is undesirable, as for the recognition of self-antigens and the maintenance of steady state. Understanding this process has become the focus of interest for the design of therapeutic strategies to silence autoreactive T cells in autoimmune diseases.

It has been reported that tolerogenic dendritic cells (tDCs) generated from monocytes of patients with multiple sclerosis (6), type 1 diabetes (T1D) (7), or rheumatoid arthritis (RA) (8) are able to induce a stable hyporesponsive state in CD4^+^ T cells in an antigen-specific manner. In animal models of experimental autoimmune encephalomyelitis (EAE) (9) and collagen-induced arthritis (CIA) (10), inoculated tDCs induced antigen-specific T-cell anergy and thereby impeded disease progression. Furthermore, it has been reported that tDCs were capable of inducing donor-specific hyporesponsiveness and prolonging cardiac allograft survival in mouse models of transplantation (11, 12).

The current review takes a closer look at recent findings on T-cell anergy induced by tDCs and discusses the potential of T-cell anergy for clinical applications to control undesired immune responses mediated by CD4^+^ T cells.

## Tolerogenic Dendritic Cells and the Modulation of T-Cell Responses

Dendritic cells are professional antigen-presenting cells that are able to initiate and shape T-cell responses (13). Whether DCs induce T-cell immunity or tolerance is determined by their maturation state. Mature DCs are considered to be immunogenic as they display high levels of MHC-class II and costimulatory molecules on their surface (14) as well as a proinflammatory cytokine secretion profile (15), equipping them with the capacity to efficiently present antigen and provide activating signals to CD4^+^ T cells, thus promoting their polarization toward T helper (Th) type 1, Th2, or Th17 cells. In contrast, immature DCs express low levels of MHC-II and costimulatory molecules and are mainly localized in blood and non-lymphoid tissues, where they act as sentinels specialized in capturing and recognizing antigens. A small proportion of DCs, termed semi-mature DCs, undergo partial maturation under steady-state conditions, resulting in upregulation of antigen presenting and lymph node homing capacity while proinflammatory cytokine secretion remains absent (16). Both immature and semi-mature DCs are regarded as tolerogenic because of their ability to favor T-cell differentiation to IL-10-secreting cells with regulatory properties (17). There are distinct mechanisms by which tDCs prevent T-cell responses against self-antigens *in vivo*, including deletion of autoreactive T cells, deviation of the T-cell cytokine secretion profile, generation of Tregs, and/or induction of anergy (1–3, 18). During the last decade, research has focused on the *in vitro* generation of tDCs with a stable phenotype. Human DCs are generated from peripheral blood monocytes cultured in the presence of GM-CSF and IL-4, and laboratory strategies to induce a tolerogenic phenotype include the addition of cytokines, such as IL-10 or TGF-β (19); pharmacological modulation by vitamin D3, rapamycin, or dexamethasone (20); or genetic modifications, such as IL-10 gene transduction; and silencing of CD40, CD80, or CD86 expression by RNA interference (21). Additional activation of tDCs by lipopolysaccharide (LPS) or its non-toxic analog monophosphoryl lipid A (MPLA) has been shown to improve their antigen-presenting capacity and to induce the expression of chemokine receptors that enable migration to secondary lymph nodes (22). Regardless of the strategy used for their generation, tDCs exhibit common characteristics such as low expression of costimulatory molecules, a decreased antigen-presenting capacity, and an anti-inflammatory cytokine secretion profile (20, 23, 24) and have been reported to inhibit the proliferation and activation of allogeneic and antigen-specific CD4^+^ T cells (22), to promote the differentiation into IL-10-secreting Tregs (20, 25), and to render T cells anergic (19).

Additionally, generation of murine DCs from bone marrow (BMDCs) has been described using GM-CSF alone or in combination with IL-4 (26, 27) or Fms-like tyrosine kinase 3 (Flt3) (26). In a similar fashion of human DCs, a tolerogenic phenotype can be induced in murine DCs using different cytokines, pharmacological agents, or genetic modifications.

Recently, Helft and coworkers (2015) showed that the classical method to generate BMDCs using GM-CSF (28) produces heterogeneous CD11c^+^ MHCII^+^ populations that comprise conventional BMDCs, induced by GM-CSF (GM-DCs), and monocyte-derived macrophages, induced by GM-CSF (GM-Macs), that display distinct immune functions *in vitro* and *in vivo* (29). In the procedure of GM-DCs generation, many laboratories commonly employ magnetically enriched or FACS-sorted CD11c^+^ assuming incorrectly that this DC population is homogeneous and that any cell-to-cell variation is the result of different maturation state (30, 31).

Despite this discovery, the modulatory effects of *ex vivo*-generated tolerogenic BMDCs, produced under GM-CSF protocol, have been extensively studied on CD4^+^ T cells in murine models of autoimmune diseases and transplantation. These tDCs were shown to inhibit destructive immune responses in models of bone marrow and organ transplantation (32, 33) and to exert beneficial effects in mice with CIA (34, 35), diabetes (36), and EAE (37). Thus, although the classic mouse BMDCs generation protocol results in a heterogeneous population, their immune modulatory effects have been successfully demonstrated for a long time by several authors.

## T-Cell Regulation or Anergy ?

T-cell anergy is induced when negative signals outweigh the activatory signals provided by antigen-presenting cells. Originally, anergy was defined as unresponsive state induced in T cells that recognize antigen in the absence of costimulatory signals (38), usually provided by the binding of CD28 on T cells to its ligands, namely B7 molecules, expressed on DCs (39). Consequently, proliferation and cytokine production of T cells are impaired upon reencountering the same antigen (38). It has been observed that this hyporesponsive state could be reversed in the presence of IL-2 and that signaling through the IL-2 receptor prevented the establishment of anergy in the absence of costimulation, which is consistent with the *in vitro* definition of anergy (40). In contrast, the definition of *in vivo* anergy has been more difficult and presents characteristics that differ from *in vitro* induced anergy such as the failure of exogenous IL-2 to reverse the anergy state (41).

Anergy can also be induced by coinhibitory signals through CTLA-4 (cytotoxic T lymphocyte-associated protein 4) or PD-1 (programmed cell death 1) receptors (42–44). CTLA-4 interacts with B7 molecules, preferentially with CD80, while PD-1 binds to PD-L1 and PD-L2 ligands on DCs. Moreover, tissue-derived adenosine, acting via the adenosine A2A receptor (A2AR), represents another important negative regulator of T-cell activation, able to promote long-term anergy even in the presence of costimulation (45).

Further studies show that anergy induction and maintenance depend on the presence of “anergy-associated factors” (2) such as GRAIL (gene related to anergy in lymphocytes), Cbl-b (Casitas B-cell lymphoma-b), and Itch (itchy homologue E3 ubiquitin protein ligase) (2), as well as the transcription factors Egr (early growth response) type 2 and 3 (46). GRAIL, CbI-b, and Itch are E3 ubiquitin ligases involved in cell signaling and protein ubiquitination and are modulated via the calcium/calcineurin pathway (47, 48).

Gene expression studies performed after TCR stimulation in the presence or absence of costimulation revealed upregulation of GRAIL in anergic CD4^+^ T cells (49). The role of CbI-b was identified by comparing the proliferative response of peripheral T cells from Cbl-b knockout mice and wild-type mice. Peripheral T cells from Cbl-b knockout mice hyperproliferated (50), suggesting that loss of Cbl-b impairs the induction of a T-cell hyporesponsive state associated with tolerance (51). T cells from Itch-deficient mice were shown to be resistant to anergy induction, sustaining the role of Itch in the promotion of a hyporesponsive state (47).

Egr2 was demonstrated to be the major transcription factor for anergy induction both *in vitro* and *in vivo* (52), and its overexpression was shown to inhibit T-cell activation (46, 53). Egr2 and Egr3 direct the expression of anergy-inducing genes either in cooperation with the transcription factor NFAT (nuclear factor of activated T cells) (48) or in an independent manner. The proteins encoded by those Erg-regulated genes (e.g., Grail, Cbl-b, and Itch) are required to induce a functional unresponsiveness state through downregulation of TCR signaling by inactivation or degradation of signaling molecules (54).

It has been observed that some of the anergy-associated factors and pathways are also involved in the generation of Tregs (55) (Table 1). For example, GRAIL is up-regulated in CD4^+^CD25^+^ Tregs too, and its expression is linked to their regulatory activity (56). Cbl-b and Itch also regulate the development of Foxp3^+^ Tregs in the periphery by modulating key components of TCR and TGF-β signaling pathways (57). Moreover, Egr2 is a central transcription factor for IL-10-secreting regulatory T cells expressing lymphocyte activation gene 3 (LAG-3) (58). Likewise, it has been reported that NFAT proteins are not only involved in the induction of T-cell anergy (59) but also mediate the suppressive function of Tregs by forming a cooperative complex with Foxp3 (60). Finally, A2AR signaling has been shown to induce T-cell anergy as well as Foxp3^+^ and LAG-3^+^ Tregs *in vivo* (45).

**Table 1:** **Comparison of anergic T cells and regulatory T cells**.

	Anergic T cells versus regulatory T cells	
Characteristic transcription factor	Egr2	Foxp3
Suppressor activity	Controversial	Yes
Cytokines	None/IL-10	IL-10, TGF-β
Proliferative responses	No	Yes
Role of costimulation	Absence required	Is required
Stability	Stable in the presence of the specific antigen	Stable; plasticity under certain conditions
Shared phenotype markers	GRAIL	
	Cbl-b	
	Itch	
	CTLA-4	
	LAG-3	

At the same time, it has been reported that anergic T cells can also acquire functions of Tregs. In an *in vivo* model of peripheral tolerance, antigen-specific anergic T cells were shown to secrete high levels of IL-10, suggesting that these anergic cells could act as Tregs (61). Steinbrink observed that anergic T cells, induced by IL-10-treated DCs, were able to suppress the activation and function of T cells in an antigen-specific manner (62). In this model, suppression was linked to CTLA-4-dependent cell cycle arrest (63). In another study, Pletinckx and colleagues showed that immature DCs were capable of converting anergic CD4^+^ T cells into Foxp3-IL10^+^ Tregs through engagement of CD28 and CTLA-4 (64).

T-cell anergy and Tregs induction are crucial mechanisms for the reestablishment of tolerance (9, 10, 65, 66), and although presenting different phenotypic and functional characteristics (Table 1), both mechanisms have in common the expression regulation of some genes, such as *Pd-1* (67, 68), *Icos* (55), *Lag3* (55), *Ctla-4* (55, 67), *Egr2* (55, 67), *Grail* (49, 56), *Cbl-b* (57), and *Itch* (57). Regarding the therapeutic potential of strategies inducing either anergy or Tregs, the question arises whether one or the other mechanism is more effective. Due to their capacity to efficiently suppress effector T-cell responses, Tregs were assumed to be the protagonists in tolerance induction. Deficiency or altered function of Tregs is associated with increased severity and activity of autoimmune disease (69). However, there is evidence that Foxp3^+^ Tregs may convert into proinflammatory Th17 cells in a proinflammatory cytokine environment (70, 71). This plasticity or instability of Tregs is a disadvantage for their therapeutic application. On the other hand, the induction of a hyporesponsive state in T cells has proven to be stable and autoantigen-specific, enabling silencing of self-reactive T cells in autoimmune diseases (9, 10, 72–74). The possibility that these anergic T cells can acquire suppressive capacities would strengthen their therapeutic potential to control undesired immune responses.

## Therapeutic Potential of T-Cell Anergy

The assumption that rendering autoreactive cells hyporesponsive might be a strategy to reestablish tolerance in conditions of autoimmunity and transplantation has prompted several preclinical studies to evaluate this approach in rodent models of multiple sclerosis, T1D, RA, and transplantation.

It has been previously reported that tDCs, modulated with vitamin D3 and loaded with myelin peptides, induce hyporesponsiveness of autologous myelin-specific T cells from multiple sclerosis patients *in vitro* (6). Mansilla and colleagues demonstrated the preventive and therapeutic effect of administering vitamin D3-modulated BMDCs stimulated with LPS and pulsed with encephalitogenic myelin oligodendrocyte glycoprotein (MOG) peptide *in vivo* in the EAE mouse model (9). Splenocytes from mice that received those tDCs showed reduced MOG-specific proliferation and increased IL-10 production. Another study by Zappia and coworkers reported that administration of mesenchymal stem cells (MSCs), multipotent stromal cells with immunomodulatory properties, ameliorated EAE through the induction of T-cell hyporesponsiveness (72). In this model, MSCs inhibited the proliferative response of T cells from spleen and lymph nodes to MOG peptide and polyclonal stimuli, without increasing the frequency of Tregs. In accordance with previous reports, T-cell anergy was abrogated upon administration of IL-2 (31).

Regarding T1D, it has been shown that tDCs modulated with IL-10 and TGF-β and loaded with the pancreatic islet autoantigens insulin or glutamic acid decarboxylase 65 (GAD65) were able to induce antigen-specific hyporesponsiveness in CD4^+^ T cells from patients *in vitro* (7). In a transfer model of T1D, administration of vitamin D3-treated DCs, loaded with the disease-relevant antigen BDC2.5 mimotope, induced antigen-specific hyporesponsiveness of autoreactive CD4^+^ T cells *in vitro* and *in vivo* (73). Using a transgenic mouse model of T1D, based on the concomitant expression of influenza hemagglutinin (HA) in β cells of the pancreas (under control of the insulin promoter) and of an HA-specific MHC class II-restricted TCR, it has been demonstrated that myeloid-derived suppressor cells (MDSCs) pulsed with HA peptide, effectively suppressed HA-specific T-cell responses against pancreatic islet cells and thus prevented the development of diabetes. In this study, Gr-1^+^CD115^+^ MDSCs were obtained from syngeneic colon cancer MCA26 and from syngeneic lung carcinoma (74). The beneficial effect exerted by MDSCs involved the induction of T-cell hyporesponsiveness and the generation of Tregs.

Concerning RA, Harry and coworkers showed that tDCs from healthy donors and RA patients generated in the presence of dexamethasone, vitamin D3, and MPLA, and loaded with tuberculin purified protein derivative (PPD), induced only poor antigen-specific proliferation and production of IFN-γ and IL-17 by autologous T cells, even when T cells were previously primed by PPD-loaded mature DCs (8). In a mouse model of CIA, the same investigators showed that semi-mature BMDCs modulated with dexamethasone, vitamin D3, and LPS, and pulsed with the arthritogenic antigen collagen type II (CII), migrated to the inflamed articulation and reduced progression of arthritis (75). In this model, injection of those tDCs led to diminished CII-specific proliferation within splenocytes and decreased numbers of pathogenic Th17 cells while increasing the proportion of IL-10-producing CD4^+^ T cells. In another study performed by Popov and coworkers, the administration of tDCs, modulated with the NF-κB inhibitor LF 15-0195 and pulsed with CII, delayed the onset of CIA and reduced the severity of the disease through the conversion of CII-specific T cells to a hyporesponsive state (10).

In a mouse model of transplantation, Fas ligand (FasL)-transfected murine BMDCs, displaying a tolerogenic phenotype, were able to inhibit allogeneic mixed leukocyte reaction *in vitro* and induced alloantigen-specific hyporesponsiveness *in vivo* dependent on FasL/Fas receptor interaction (11). The transfer of FasL-transfected tDCs significantly prolonged the survival of fully MHC-mismatched vascularized cardiac allografts by favoring the development of alloantigen-specific hyporesponsiveness (11). Another study demonstrated that dexamethasone-modulated and LPS-activated tDCs induce donor-specific T-cell hyporesponsiveness against the allograft and thereby prolong survival of cardiac allografts (12).

These *in vivo* studies support the suitability of strategies to induce antigen-specific T-cell anergy for the reestablishment of tolerance in patients with autoimmune disorders or transplants.

Currently, a number of clinical trials are being conducted. Giannoukakis et al. demonstrated safety of tDCs in T1D patients (76). In the study by Benham et al., tDCs from RA patients generated with BAY11-7082 and pulsed with citrullinated peptides showed a significant reduction of IL-6 response to vimentin in *ex vivo* antigen-specific T-cell proliferation assays. Effector T cells decreased after treatment and the underlying mechanism might include deletion or anergy in response to antigen recognition (77). Another study by Harry et al. is intended to assess safety, feasibility, and acceptability of Dex-VitD3-treated tDCs therapy (8). Additionally, the ability to modify antigen-specific pathogenic responses is also being evaluated using vaccines of synthetic peptides representing T-cell epitopes, such as Hsp90 on T1D patients (78), contributing to preservation of β-cell function and glycemic control, and dnaJP1 on RA patients (79), showing a reduction in the percentage of TNF-producing T cells.

Further mechanistic studies are needed in order to determine the efficacy of antigen-specific therapies for autoimmunity and the role of T-cell anergy.

## Concluding Remarks

Suppression of antigen-specific T-cell responses either through the expansion of Tregs or the induction of anergy represents an attractive immunotherapeutic approach to target autoreactive T cells in autoimmune diseases. Despite the differences, both tolerance mechanisms share some fundamental signaling pathways and regulate the expression of common genes. The generation of Tregs has hitherto been the focus of interest; however, Tregs can exert unspecific regulation and may be prone to conversion into proinflammatory Th17 cells. In contrast, the induction of a stable hyporesponsive state appears to be a promising strategy to specifically silence self-reactive T cells in autoimmune diseases without undesired adverse effects. *In vitro* experiments confirmed that anergy induction efficiently prevents responses against disease-associated autoantigens in CD4^+^ T cells of patients with autoimmune pathologies, including multiple sclerosis, T1D, or RA. *In vivo*, anergy induction in autoreactive CD4^+^ T cells has been proven to control disease onset and progression in murine models of autoimmune diseases. The possibility that anergic T cells can also acquire suppressive capacities supports their fundamental role in the control of immune responses. Thus, T-cell anergy is an effective mechanism to eradicate aberrant T-cell responses to “self” and its induction by tDCs provides a promising therapeutic strategy for the reestablishment of self-tolerance in patients with autoimmune diseases.

## Conflict of Interest Statement

The authors declare that the research was conducted in the absence of any commercial or financial relationships that could be construed as a potential conflict of interest.
